# Ovarian cancer stem-like side-population cells are tumourigenic and chemoresistant

**DOI:** 10.1038/sj.bjc.6605626

**Published:** 2010-03-30

**Authors:** L Hu, C McArthur, R B Jaffe

**Affiliations:** 1 Center for Reproductive Sciences, Department of Obstetrics, Gynecology and Reproductive Sciences, University of California, San Francisco, CA 94143–0556, USA; 2 Sandler Sorting Facility, Department of Medicine, University of California, San Francisco, CA 94143–0556, USA

**Keywords:** side population, ovarian cancer stem-like cells, tumourigenesis, drug resistance, ABCG2

## Abstract

**Background:**

Ovarian cancer is the most lethal gynaecological malignancy. Although ovarian cancer patients often respond initially to chemotherapy, they usually develop chemoresistance. We hypothesised that a small portion of ovarian cancer cells have stem-like cell properties that contribute to tumourigenesis and drug resistance.

**Methods:**

Flow cytometry and Hoechst 33342 efflux isolated side-population (SP) cells from ascites derived from ovarian cancer patients and from mice inoculated with human ovarian cancer cell lines. The SP cells were examined for stem cell markers OCT4, NANOG, STELLAR, and ABCG2/BCRP1 by immunocytochemistry and RT–PCR. The SP cells and non-SP cells were studied for tumourigenesis and chemoresistance *in vitro* and *in vivo*.

**Results:**

The SP cells expressed ABCG2/BCRP1, OCT4, STELLAR, and NANOG, detected by immunocytochemistry and RT–PCR. ABCG2/BCRP1 expression was higher in SP than in non-SP cells. Xenogeneic mice inoculated with SP cells yielded more tumours than did mice inoculated with non-SP cells. In parallel, SP cell culture resulted in extensive cell proliferation, which was markedly more than in non-SP cells. SP cells resisted chemotherapy compared with non-SP cells, both *in vivo* and *in vitro*.

**Conclusion:**

Ovarian cancer SP cells are tumourigenic and chemoresistant. ABCG2/BCRP1 has an important role in chemoresistance, which has implications for new therapeutic approaches.

The majority of ovarian cancer patients with advanced disease eventually develop chemotherapy resistance. The concept of cancer stem-like cells in solid tumours opens new approaches to carcinogenesis and chemotherapy. Cancer stem-like cells share many properties with normal stem cells: they have a protracted lifespan, relative quiescence, ability for self-renewal, the capacity to induce tumourigenesis, and resistance to chemotherapeutic agents and apoptosis (Reya *et al*, 2001; Beachy *et al*, 2004; Sales *et al*, 2007; Wu *et al*, 2007).

Surface markers expressed on cancer stem-like cells can be used to isolate these cells. However, these markers differ in different types of tumours. The capacity to extrude Hoechst dye, one of the stem cell characteristics, has also been used to identify cancer stem-like cells (Dean *et al*, 2005). These cells express drug transporters that make them resistant to many chemotherapeutic agents (Allikmets *et al*, 1998a; Dean *et al*, 2005). ABCG2/BCRP1 (breast cancer-resistance protein-1), an ATP-binding cassette (ABC) transporter, is a cell surface drug-resistance marker as well, which has been used to identify stem cells from a variety of tissues, including tumours (Zhou *et al*, 2001b; Diestra *et al*, 2002a). ABCG2/BCRP1 expression confers resistance to chemotherapeutic agents and permits cells to exclude Hoechst dye 33342 (Molecular Probes, Eugene, OR, USA) (Allikmets *et al*, 1998a; Dean *et al*, 2005). This dye-excluding side-population (SP) phenotype has been used in various tissues to select presumptive stem-like cells. Side-population cells are enriched with cancer stem-like cells in a variety of carcinomas (Kondo *et al*, 2004a; Hirschmann-Jax *et al*, 2005; Haraguchi *et al*, 2006; Ho *et al*, 2007; Wang *et al*, 2007). These cells have been found in several tissues and cell lines, including bone marrow, skin, lung, mammary epithelium, and embryonic stem cells (Alvi *et al*, 2003; Nishimura *et al*, 2003; Majka *et al*, 2005; Yano *et al*, 2005). They have been isolated from malignancies, including leukaemia (Feuring-Buske and Hogge, 2001; Wulf *et al*, 2001) and breast (Doyle and Ross, 2003), brain (Hirschmann-Jax *et al*, 2005), prostate (Hirschmann-Jax *et al*, 2005), retinoblastoma (Seigel *et al*, 2005), lung (Giangreco *et al*, 2004), and ovarian (Patrawala *et al*, 2005; Szotek *et al*, 2006) cancers.

The SP phenotype, coupled with the expression of stem cell markers, is the hallmark of normal stem cells and cancer stem-like cells as well (Zhou *et al*, 2001b; Scharenberg *et al*, 2002; Diestra *et al*, 2002a; Dean *et al*, 2005).

We explored the tenet that SP cells isolated from human ovarian cancer cells by Hoechst dye exclusion after flow cytometry show stem cell characteristics. The SP cells were examined for the drug-resistance transporter, ABCG2/BCRP1, and for other stem cell markers, OCT4, NANOG, and STELLAR, by immunocytochemistry and RT–PCR. The SP and non-SP cells were studied for tumourigenesis and chemoresistance *in vitro* and *in vivo*.

## Materials and methods

### Materials

The ABCG2/BCRP1 monoclonal antibody (BXP-21) was from Sigma Chemicals (St Louis, MO, USA). The OCT 4 monoclonal antibody was from Chemicon (Tarrytown, NY, USA). NANOG and STELLAR polyclonal antibodies were kindly provided by Dr A. Clark (University of California, San Francisco (UCSF)). Cisplatin was from Sigma Aldrich (St Louis, MO, USA). The human OVCAR3 cell line was a gift from Dr T. Hamilton (Fox Chase Cancer Center, Philadelphia, PA, USA), and the ovarian cancer cisplatin-resistant cell line, A2780-CP, was a gift from Dr J. Chan (UCSF). All other cell lines were from the UCSF Gynecologic Ovarian Tissue Bank. Culture reagents were obtained from the Cell Culture Facility, UCSF.

### Human ovarian cancer ascites

Primary ovarian cancer cells were collected from stage III fresh ovarian cancer patient ascites at laparotomy through the UCSF Gynecologic Ovarian Tissue Bank. Ascite fluid was collected and was placed in a refrigerator at 4 °C for 1–2 h. After discarding the supernatant, cells were resuspended in medium (RPMI-1640) and centrifuged to isolate the cellular components. The cells were then cultured with medium RPMI-1640 supplemented with 2.0 g l^–1^ glucose and 0.3 g l^–1^ L-glutamine, as well as with 10% fetal bovine serum (FBS), 1% penicillin/streptomycin, and 1% fungizone and incubated at 37 °C. Cells were analysed within 7 days, during which sorting of SP cells by flow cytometry, RT–PCR, and immunoassay was performed.

### Experimental animals

Female athymic immunodeficient mice (Simonsen Laboratories, Gilroy, CA, USA) were delivered to the UCSF Laboratory Animal Resource Center, and were housed in isolated conditions, fed autoclaved standard pellets and water, and allowed to adapt to their new environment. All protocols involving immunodeficient mice were approved by the Committee on Animal Research, UCSF.

### Mouse ovarian cancer ascites

Mouse ascites were collected from female athymic nude mice (5–7 weeks old) inoculated i.p. with human OVCAR3 cells for 6 weeks. Cells were analysed within 7 days with sorting of SP cells by flow cytometry, and RT–PCR and immunoassay were performed as described in the human ovarian cancer ascites section.

### Identification of SP cells

Identification of SP cells was performed as described previously by Goodell (2002). Cells were incubated with 5 *μ*g ml^–1^ Hoechst 33342 dye (Molecular Probes) for 90 min with and without 50 *μ*M verapamil. Cell samples were analysed and sorted using a Moflo MLS cell sorter (Beckman-Coulter, Inc., Hialeah, FL, USA) with UV capabilities and SUMMIT software for data acquisition and analysis. An argon laser was used to excite the Hoechst dye. Fluorescence emission was collected with a 405/30 nm bandpass filter for Hoechst blue and a 670/40 nm bandpass filter for Hoechst red. Dead cells were excluded by propidium iodide fluorescence at 670/30 nm.

### Kidney capsule graft

Side population cells or non-SP cells (500 cells each) were isolated from ascites derived from mice inoculated with the human ovarian cancer cell line, OVCAR3, or from ascites derived from ovarian cancer patients, suspended in 50 *μ*l of rat tail collagen gel as previously described (Hayward *et al*, 2001) and grafted under the kidney capsule of female athymic mouse hosts. The hosts were killed after 8 weeks of growth and the kidneys with grafts were explanted, fixed in formalin, and embedded in paraffin.

### Intraperitoneal inoculation of SP cells

Two groups of nude mice (*n*=12) were inoculated separately i.p. with SP cells *vs* non-SP cells (20 000 cells each) from A2780-CP (cisplatin-resistant human ovarian cancer cell line). At 8 weeks after inoculation, the mice were killed and tumour burden was quantified. In a separate experiment, four groups of nude mice (5–7 weeks old, *n*=16) were inoculated i.p. with SP *vs* non-SP cells (20 000 cells) from A2780-CP. At 2 weeks after inoculation, two groups (SP (*n*=4) *vs* non-SP cells (*n*=4)) were treated with cisplatin (10 mg kg^–1^ B.W.) 3 times weekly for 3 weeks. Two additional groups (SP (*n*=4) *vs* non-SP cells (*n*=4)) received vehicle alone.

#### SP culture

Side population cells and non-SP live cells (500 cells each) obtained from ascites derived from ovarian cancer patients were seeded on 24-well plastic culture plates. Cells were cultured in RPMI-1640 containing 10% FBS, 100 units ml^–1^ penicillin, and 100 *μ*g ml^–1^ streptomycin. Cultures were incubated at 37 °C in a humidified atmosphere of 95% O_2_ and 5% CO_2_. The medium was changed every other day. The morphological appearance of cells was captured at the end of the fifth day of culture. At least ten × 150 microscopic fields were scored.

#### Reverse transcription–polymerase chain reaction (RT–PCR)

Total RNA was extracted from pellets of human ovarian cancer cells from ascites derived from patients and mice bearing human OVCAR3 cells using an RNeasy minikit (Qiagen, Hiden, Germany) according to the manufacturer's protocol. Reverse transcription–polymerase chain reaction was then performed using a OneStep RT–PCR Kit (Qiagen). The primers used for PCR amplification were BCRP1 (231 bp): 5′-CAACCATTGCATCTTGGCTG-3′ and 5′-CAAGGCCACGTGATTCTTCC-3′ STELLAR (174 bp): 5′-GTTACTGGGCGGAGTTCGTA-3′ and 5′-TGAAGTGGCTTGGTGTCTTG-3′. Conditions for both BCRP1 and STELLAR were 95 °C for 1 min, 58 °C for 1 min, and 72 °C for 2 min, for 40 cycles. Acquired DNA samples were loaded onto a 2% agarose gel and analysed.

#### Fluorescent immunostaining in suspension

Patient ovarian cancer cells or OVCAR3 cells were double labelled in suspension with Hoechst 33342, and with either ABCG2/BCRP1 or OCT4. Cells (2 × 10^6^) were centrifuged at 800 r.p.m. for 5 min. The supernatant was decanted and cells were resuspended in 4 ml RPMI with 10% fetal calf serum. Hoechst 33342 dye (Molecular Probes) was added at 5 *μ*g ml^–1^, and cells were incubated at 37 °C for 90 min. Cells were washed twice in PBS and centrifuged for 5 min. Cells were resuspended in PBS and then received 1 : 200 ABCG2/BCRP1 antibody or OCT 4 antibody and were incubated for 1 h at room temperature. Cells were washed twice with PBS and resuspended. TRITC-conjugated anti-mouse IgG (Sigma Chemicals) was added at 1 : 100 dilution. Cells were incubated for 1 h at room temperature. After washing twice with PBS, cells were resuspended and pipetted onto a slide and covered with a coverslip for microscopic viewing.

#### Immunofluorescent analysis of SP cells

Side population cells were sorted directly onto poly-L-lysine-coated slides, then air-dried and fixed in 50 : 50 methanol/acetone for 5 min as described by Alvi *et al* (2003). The cells were pretreated with 3% horse serum in PBS plus 0.05% Tween-20 for 1 h, and then rinsed in PBS and incubated with both 1 : 500 rabbit anti-NANOG antibody and 1 : 200 mouse anti-ABCG2/BCRP1 antibody for 1 h. The cells were thoroughly washed in PBS before application of 1 : 200 of both FITC-conjugated anti-rabbit IgG and TRITC-conjugated anti-mouse IgG, and finally washed again in PBS. A similar procedure was used for staining CD34 1 : 200 with mouse anti-CD34 antibody and for staining CD44 1 : 200 with mouse anti-CD44 antibody.

#### Immunofluorescence in tumour tissues

The ovarian cancer tissue from mice inoculated with OVCAR3 cells was washed extensively in PBS. Before immunostaining, tissues were pretreated with 3% horse serum in PBS plus 0.05% Tween-20 for 1 h. Tissues were rinsed in PBS and incubated with both 1 : 500 rabbit anti-NANOG antibody and 1 : 200 mouse anti-ABCG2/BCRP1 antibody for 16 h on an orbital shaker at 4 °C. Tissues were washed thoroughly in PBS before application of 1 : 200 of both FITC-conjugated anti-rabbit IgG and TRITC-conjugated anti-mouse IgG, and finally washed again in PBS. Immunostained tissues were examined by microscopy.

#### Apoptosis assessment

Side population cells *vs* non-SP cells were seeded (5000 cells) on eight-well glass culture slides. Cells were cultured in RPMI-1640 for 24 h. The medium was then removed and replaced with culture medium in four individual wells for SP cells *vs* non-SP cells: (1) vehicle, (2) cisplatin 5 *μ*M, (3) verapamil 50 *μ*M, and (4) cisplatin plus verapamil, for an additional 48 h. After fixing with 2% paraformaldehyde, cells were used to assess apoptosis. DNA labelling with digoxigenin dUTP and terminal transferase, followed by immunocytochemical staining with peroxidase-coupled antidigoxigenin antibody and diaminobenzidine, was carried out with the reagents supplied in the Apoptag kit (Intergen, Purchase, NY, USA). After light counterstaining with haematoxylin, nuclei that stained brown were scored as positive for apoptosis and those that stained blue were scored as negative. At least ten × 150 microscopic fields were scored, and the apoptotic index was calculated as the percentage of cells that were scored positive.

#### Light microscopy and analysis

Ovarian cancer tissue and cells immunostained with ABCG2/BCRP1, OCT4, and NANOG were examined with a Leica DMRB or Leica (Allendale, NJ, USA) Ortholux II photomicroscope at low and high magnification.Images were collected with a Photonics (Fukuoka, Japan) DEI-470 CCD camera and a RasterOps (Santa Clara, CA, USA) 24X LTV frame grabber, imported directly into Adobe Photoshop (San Jose CA, USA). Photomicrographic plates were composed from the original data in Photoshop without alteration or manipulation.

#### Statistics

Data were analysed using the unpaired Student's *t-*test for comparison between groups. Differences between groups were considered statistically significant at *P*<0.05. Experiments *in vivo* were performed in duplicate, whereas experiments *in vitro* were performed in triplicate.

## Results

### Ovarian cancer SP cells

**Figure 1:**
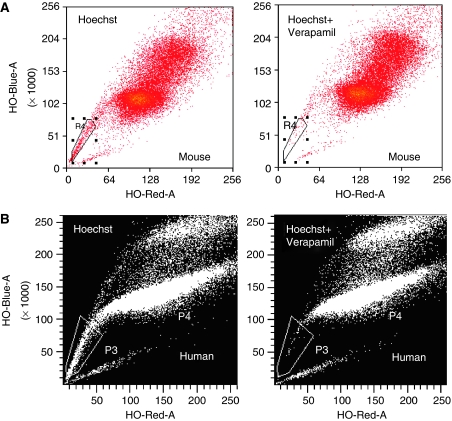
(**A**) Side population (SP) of human OVCAR3 ovarian cancer cells from mouse ascites detected by flow cytometry. Boxed cells (labelled R4) indicate Hoechst-excluding SP. (**B**) SP of patient ascites-derived ovarian cancer cells detected by flow cytometry. Boxed cells (labelled P3) indicate Hoechst-excluding SP. Addition of 50 *μ*M verapamil resulted in a marked reduction in SP in **A** and **B**.

Figure 1 shows two representative experiments from flow cytometry. Panel A shows a small number of cells (1.65%) in the box R4 from the SP from OVCAR3 cells. The SP was blocked by 88% by 50 *μ*M verapamil. Panel B illustrates a small number of cells (0.3%) in the box P3 from the SP from human ovarian cancer cells. The SP was blocked 80% by 50 *μ*M verapamil.

The mean percentage of SP cells from ascites derived from human ovarian cancer patients, ascites from mice inoculated with OVCAR3, and A2780-CP cell lines were 0.4±0.05%, 1.01±0.27%, and 0.45±0.05%, respectively. We also sorted SP cells from other human ovarian cancer cell lines, including A2780, HEYA8, OCC1, and SKOV3. Percentages from these cell lines ranged from 0.1 to 2.4%, (A2780: 0.2% HEYA8: 2.4% OCC1: 0.1% and SKOV3: 1.7%).

### OCT4 and ABCG2/BCRP1 immunopositive cells colocalise with Hoechst-dim cells in OVCAR3 cells

**Figure 2:**
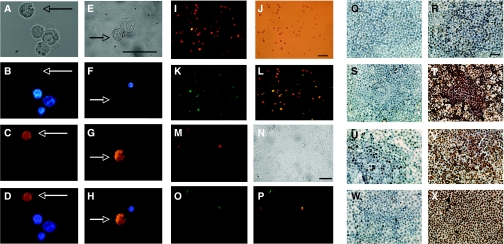
(**A–D**) Colocalisation of cells from mouse ascites bearing human ovarian cancer cells with OCT4 and Hoechst dye 33342. (**E–H**) Colocalisation of cells from mouse ascites bearing human ovarian cancer cells with ABCG2/BCRP1 and Hoechst dye 33342. (**A, E)** Bright-field; (**B, F**) Hoechst-dye uptake (blue); (**C**) OCT4 (bronze); (**G**) ABCG2/BCRP1 (bronze); (**D**) merged image **B** and **C**; and (**H**) merged image **F** and **G**. Arrows indicate the cells that are immunoreactive for OCT4 or ABCG2 and Hoechst-dim. Bar =10 *μ*m. (**I–L**) Immunolocalisation of ABCG2/BCRP1 (red fluorescence) and NANOG (green fluorescence) in side population of human ovarian cancer cells (OVCAR3) derived from mouse ascites. (**I**) ABCG2/BCRP1; (**J**) bright-field; (**K**) NANOG; (**L**) merged **I** and **J**. Bar =10 *μ*m. (**M**–**P**) Immunolocalisation of ABCG2/BCRP1 (red fluorescence) and NANOG (green fluorescence) in mouse cancer tissues bearing human ovarian cancer cells. (**M**) ABCG2/BCRP1; (**N**) bright-field; (**O**) NANOG; and (**P**) merged **M** and **O**. Arrows indicate cells that are immunoreactive for OCT4 or ABCG2. Bar =10 *μ*m. (**Q**–**X**) SP *vs* non-SP cell apoptosis *in vitro*. Apoptosis of SP (**Q**–**T**) *vs* non-SP cells (**U**–**X**) after 48-h treatment with cisplatin and/or verapamil *in vitro*. (**Q**, **U**) Control; (**R**, **V**) cisplatin; (**S**, **W**) verapamil; and (**T**, **W**) cisplatin plus verapamil. Brown nuclei indicate apoptotic cells. Scale bar=10 *μ*m. Note that SP cells are resistant to cisplatin (**R**), but this resistance is reversed by verapamil (**T**).

Staining of OCT4-immunopositive OVCAR3 cells was detected in Hoechst-dim cells, further suggesting a stem cell phenotype (Figure 2A–D). OVCAR3 cells that were Hoechst-dim (Figure 2B) were OCT4-bright (Figure 2C), as seen more definitively in the merged image (Figure 2D). Bright-field images were captured (Figure 2A) to ensure that labelled cells appeared intact.

Figure 2E–H shows that ABCG2/BCRP1 confers the ability to exclude Hoechst dye. OVCAR3 cells immunoreactive for ABCG2/BCRP1 were Hoechst-dim (Figure 2F and G). OVCAR3 cells that were ABCG2/BCRP1-bright were Hoechst-dim, as seen in the merged image (Figure 2H). Bright-field images were captured (Figure 2E). These findings show that ABCG2/BCRP1 colocalises with Hoechst-dim cells.

### Immunoreactivity of stem cell markers in sub-populations of OVCAR3 cells

The SP phenotype is thought to occur through action of the ABCG2/BCRP1 transport cassette protein (Zhou *et al*, 2001a; Ahmed-Belkacem *et al*, 2006). To analyse whether SP cells coexpressed stem cell markers ABCG2/BCRP1 and NANOG, we examined their immunological activity. We found that SP cells stained strongly with ABCG2/BCRP1 antibody (Figure 2I), and that SP cells also costained with NANOG (Figure 2K), as seen particularly in the merged image (Figure 2L). Bright-field images were captured in Figure 2J. In contrast, SP cells were not stained by CD34, a haematopoietic stem cell marker (data not shown), suggesting that the preparations were not contaminated with haematopoietic stem cells. The SP cells were not stained significantly by CD44 either (data not shown).

### Cancer stem cell niche

Figure 2M–P shows the cancer stem-like cell niches in the tumour tissue. These cells share the SP phenotype and are costained with ABCG2 and NANOG, as shown in Figure 2I–L. Figure 2M shows that cells stained strongly with the ABCG2/BCRP1 antibody and also costained with NANOG (Figure 2O), as seen particularly in the merged image (Figure 2P). Bright-field images are seen in Figure 2N.

### Chemoresistance and apoptosis

Figure 2Q–T illustrates apoptosis of cultured SP cells from A2780-CP after 48 h of treatment with cisplatin and/or verapamil *in vitro*. Apoptosis in SP cells in the cisplatin-treated group was <20%, indicating that SP cells were resistant to chemotherapy. However, over 95% of SP cells in the cisplatin plus verapamil-treated group were apoptotic. There were no significant apoptotic cells in control SP and verapamil-treated SP cells. The brown nuclei and debris reflect cells that have undergone apoptosis. At least ten × 150 microscopic fields were scored.

In a parallel study, we also showed apoptosis in cultured non-SP cells in Figure 2U–X. Apoptosis of non-SP cells in both the cisplatin-treated group and cisplatin plus verapamil-treated group was over 90% of the total cell population. However, there were no significant changes in the control and verapamil-treated groups.

### Gene expression

**Figure 3:**
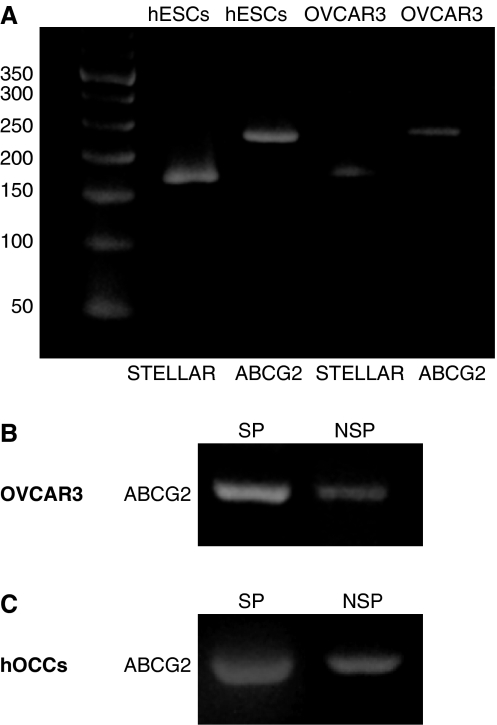
(**A**) RT–PCR analysis of ABCG2 and STELLAR in mouse ascites from human ovarian cancer (OVCAR3) and human embryonic stem cells (hESCs). (**B**) RT–PCR analysis of ABCG2 in non-SP and SP from human ovarian cancer cells (OVCAR3). (**C**) RT–PCR analysis of ABCG2 in non-SP and SP cells from human ovarian cancer cells (hOCCs).

Reverse transcriptase–PCR revealed that ABCG2/BCRP1 and STELLAR mRNA expressions were found in both human embryonic stem cells and OVCAR3 cells (Figure 3A). Because ABCG2/BCRP1, a drug-resistance transporter, is a key molecule in SP cells, ABCG2/BCRP1 mRNA was analysed in both sorted SP and non-SP cells. ABCG2/BCRP1 was expressed in SP cells more extensively than in non-SP cells (Figure 3B). ABCG2/BCRP1 expression was also found in human ovarian cancer cells from patient ascites (Figure 3C) and was expressed in SP cells more extensively than in non-SP cells.

### SP and tumourigenesis

**Figure 4:**
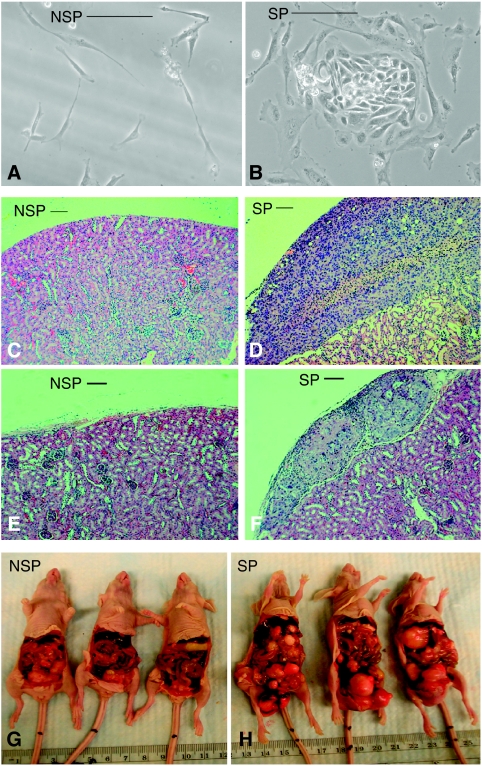
Non-SP cells (**A**) *vs* SP cells (**B**) from patient's ovarian cancer ascites were cultured for 5 days. Non-SP cells (**C**) and SP cells (**D**) from human ovarian cancer cells (500 cells) derived from patient's ascites were grafted under kidney capsules in each of two mice for 8 weeks. Non-SP cells (**E**) and SP cells (**F**) from OVCAR3 cells (500 cells) derived from mouse model ascites were grafted under kidney capsules in each of two mice for 8 weeks. Non-SP cells (**G**, three mice) and SP cells (**H**, three mice) from A2780-CP (cisplatin resistant) cells were injected i.p. into six mice (20 000 cells per mouse) for 8 weeks. Bar=10 *μ*m.

Figure 4A and B shows non-SP and SP live cells obtained from patient ovarian cancer ascites and cultured for 5 days. SP cells (Figure 4B) have high-density chromatin and undergo extensive proliferation (Figure 4A), whereas non-SP (Figure 4B) cells do not proliferate significantly.

Figure 4D shows tumour growth from SP cells derived from patient ovarian cancer ascites and grafted under the kidney capsule. In contrast, tumours did not develop in non-SP cells grafted under the kidney capsule (Figure 4C). Figure 4F illustrates tumour growth from the SP cells derived from mice inoculated with OVCAR3 cells and grafted under the kidney capsule. In contrast, tumours did not develop in non-SP cells similarly grafted (Figure 4E).

Figure 4G and H shows tumour burden in mice after 8 weeks of inoculation with non-SP or SP cells from the A2780-CP cell line injected i.p. (three representative mice in each group). Note the extensive tumourigenesis in SP-inoculated mice compared with non-SP cell-inoculated mice. The mean tumour burden in SP-inoculated mice (7.29±0.73) was significantly (*P*<0.0001) greater (93.85%) than that in non-SP-inoculated mice (0.44±0.12).

### Chemoresistance in the mouse model

**Figure 5:**
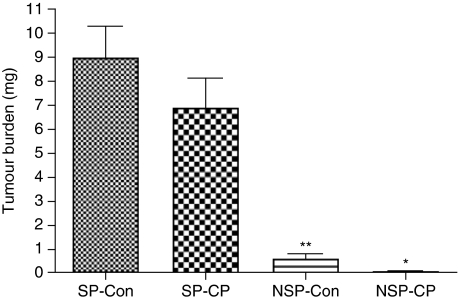
Effect of cisplatin on tumour burden in mice inoculated i.p. with SP *vs* non-SP isolated from A2780-CP cells. Treatments began 8 weeks after inoculation. Treatment groups consisted of control (vehicle) and cisplatin alone. At the end of the experiment (3 weeks of treatment), the mice were killed. At autopsy, tumours were excised and weighed. SP-Con (control)=side population without treatment; SP-CP=side population with cisplatin treatment; NSP-Con=non-side population without treatment (control); NSP-CP=non-side population with cisplatin treatment; ^*^P<0.05 *vs* NSP-CP; ^**^P<0.01 *vs* SP-CON. Data are expressed as the mean (bars±s.d.).

The effects of cisplatin on tumour growth in nude mice (*n*=16) are shown in Figure 5. Treatments began 2 weeks after the mice were inoculated with SP *vs* non-SP from A2780CP cells. Tumour burden in SP cisplatin-treated mice (6.920±1.226 g) was not significantly reduced (23.08%) compared with that in SP control mice (8.997±1.297 g), indicating that SP cells are resistant to chemotherapy. Tumour burden in cisplatin-treated mice (0.0325±0.018 g) inoculated with non-SP cells was reduced by 94.9% (*P*<0.05) compared with that in non-SP-inoculated control mice (0.6426±0.23 g). Regarding the differences in tumour burdens between SP- and non-SP-inoculated mice, tumour burden in SP control mice was greater (92.3%) than that in the non-SP control group.

## Discussion

In this study, we show that SP cells have an important role in tumourigenesis and drug resistance in human ovarian cancer, both *in vivo* and *in vitro*. We identified a small SP population from ovarian cancer cells derived from ascites from patients and from nude mice inoculated with the human OVCAR3 cell line, as well as from other human ovarian cancer cell lines, including A2780, A2780-CP, HEYA8, OCC1, and SKOV3. This is consistent with findings in other tumour types (Patrawala *et al*, 2005; Szotek *et al*, 2006).

Our study indicates that ovarian cancer cells are heterogeneous. SP cells are highly proliferative compared with non-SP cells; SP xenogeneic transplant mice grew more tumours than non-SP xenogeneic transplant mice, as shown from other studies (Chiba *et al*, 2008; Harris *et al*, 2008; Steiniger *et al*, 2008). SP cells have characteristics of cancer stem-like cells. They have a striking capacity to proliferate, differentiate, and undergo self-renewal, enabling them to eventuate in tumour formation and repopulate tumours after therapy (Alvi *et al*, 2003; Kondo *et al*, 2004b; Hirschmann-Jax *et al*, 2005). This small population is likely to be responsible for tumourigenesis. Although current chemotherapy for ovarian cancer eradicates most tumour cells with high initial response rates, the majority of patients with advanced disease eventually become resistant to chemotherapy. This raises the possibility that failure to eradicate ovarian cancers may be because of failure to treat the definitive target: cancer stem-like cells. Both our *in vitro* and *in vivo* experiments show that SP cells are resistant to chemotherapy. This novel finding suggests that SP cells contribute to drug resistance and might be an attractive target for cancer therapy.

Our study shows that ovarian cancer SP cells express the embryonic stem cell markers, NANOG, OCT4, STELLAR, and ABCG2/BCRP1. OCT4 and NANOG are transcription factors in embryonic stem cells (Nichols *et al*, 1998; Chambers *et al*, 2003; Mitsui *et al*, 2003). Poorly differentiated tumours show overexpression of genes that are normally enriched in embryonic stem cells (Ben-Porath *et al*, 2008). NANOG and OCT4 are more significantly overexpressed in poorly differentiated tumours than in well-differentiated tumours (Ben-Porath *et al*, 2008). These genes contribute to stem cell-like phenotypes found in many tumours (Dvorak and Hampl, 2005; Wong *et al*, 2008). STELLAR, also known as developmental pluripotency associated-3 (DPPA3), is expressed in human embryonic cells. Particularly relevant is the expression of ABCG2/BCRP1, a calcium-sensitive cell surface protein that excludes Hoechst dye, conferring resistance to several chemotherapeutic agents (Diestra *et al*, 2002b; Ahmed-Belkacem *et al*, 2006). The ABCG2/BCRP1 gene was first isolated from human tumour cell lines, in which it was involved in drug resistance (Doyle *et al*, 1998; Allikmets *et al*, 1998b; Miyake *et al*, 1999). ABCG2/BCRP1 is a significant marker for Hoechst dye-extruding stem cells (Zhou *et al*, 2001a). Various types of ABC transporters, including proteins encoded by multidrug-resistance gene 1 (MDR1, p-glycoprotein), multidrug resistance-associated protein 1 (MRP1), as well as ABCG2/BCRP1, have been described. In bone marrow from *mdr1a1b*^–/–^ knockout mice, a normal percentage of SP cells was obtained, suggesting that MDR1 is not correlated with the SP cells identified by Hoechst (Uchida *et al*, 2002). MDRI and MRP1 are not major contributors to the SP phenotype in bone marrow cells (Scharenberg *et al*, 2002). MDR1 (CD44) is not a significant marker in the SP cells in our study. However, several studies indicate that the presence of the ABCG2/BCRP1 transporter is highly correlated with the SP phenotype in various cells (Hirschmann-Jax *et al*, 2005) and is downregulated in committed progenitor cells (Scharenberg *et al*, 2002; Zhou *et al*, 2002). Therefore, ABCG2/BCRP1 expression is a useful marker for positive selection of several types of cancer stem-like cells (Kim *et al*, 2002; Scharenberg *et al*, 2002; Patrawala *et al*, 2005; Ahmed-Belkacem *et al*, 2006). In this study, we show that SP cells express more ABCG2 than non-SP cells, supporting the concept that cancer stem-like cells highly express ABCG2, as seen in other tumour types (Patrawala *et al*, 2005; Abbott, 2006; Lou and Dean, 2007; Olempska *et al*, 2007). Verapamil, an ABC transporter inhibitor (Zhou *et al*, 2001a), which does not significantly inhibit the MDRI transporter (Goodell, 2002), blocked SP cells from excluding the Hoechst dye, indicating the important role of ABCG2/BRCP1 in the SP of ovarian cancer cells. Our *in vitro* study of cisplatin-resistant ovarian cancer cells (A2780-CR) indicates that SP cells are resistant to chemotherapy and verapamil reverses this chemoresistance. The results suggest that SP cells resist chemotherapy, at least partially because of overexpression of ABCG2.

Stem cell markers NANOG and ABCG2/BCRP1 were colocalised in both SP and non-SP cells, and the specific individual cells in ovarian cancer tissues, as illustrated in our study, make it reasonable to hypothesise that there are cancer stem-like cell niches in ovarian cancer. The stem cell niche is in a specific locale, within the structure in which the microenvironment maintains the ‘stemness’ of stem cells (Fuchs and Segre, 2000). The cancer stem-like cell niche may be important for maintaining asymmetric divisions and stem-like properties (Iwasaki and Suda, 2009). Interaction with the cancer stem cell niche and tumour microenvironment may lead to tumourigenesis (Howe *et al*, 1998; Sternlicht *et al*, 1999).

In summary, we have shown that ovarian cancer cells are heterogenic. The SP cells have the characteristics of cancer stem-like cells. They are both highly tumourigenic and chemoresistant. The increased expression of ABCG2/BCRP1 in SP cells is responsible for chemoresistance. These observations have potentially important implications for future therapeutic strategies that target the ovarian cancer stem-like cells.
